# Curcumin and Andrographolide Co-Administration Safely Prevent Steatosis Induction and ROS Production in HepG2 Cell Line

**DOI:** 10.3390/molecules28031261

**Published:** 2023-01-27

**Authors:** Rosaria Maria Pipitone, Rossella Zito, Giulia Lupo, Ayesha Javed, Claudia La Mantia, Gabriele Di Maria, Giovanni Pratelli, Francesca Di Salvo, Simona Fontana, Marzia Pucci, Daniela Carlisi, Stefania Grimaudo

**Affiliations:** 1 Department of Health Promotion, Mother and Child Care, Internal Medicine and Medical Specialties, University of Palermo, 90127 Palermo, Italy; 2 Department of Biomedicine, Neurosciences and Advanced Diagnostics, University of Palermo, 90127 Palermo, Italy; 3 Department of Agricultural, Food and Forest Sciences, University of Palermo, 90128 Palermo, Italy

**Keywords:** curcumin, andrographolide, liver steatosis, ROS, lipid droplets, NAFLD, FABP1, GK, PPARGC1A

## Abstract

Non-alcoholic fatty liver disease (NAFLD) is an emerging chronic liver disease worldwide. Curcumin and andrographolide are famous for improving hepatic functions, being able to reverse oxidative stress and release pro-inflammatory cytokines, and they are implicated in hepatic stellate cell activation and in liver fibrosis development. Thus, we tested curcumin and andrographolide separately and in combination to determine their effect on triglyceride accumulation and ROS production, identifying the differential expression of genes involved in fatty liver and oxidative stress development. In vitro steatosis was induced in HepG2 cells and the protective effect of curcumin, andrographolide, and their combination was observed evaluating cell viability, lipid and triglyceride content, ROS levels, and microarray differential gene expression. Curcumin, andrographolide, and their association were effective in reducing steatosis, triglyceride content, and ROS stress, downregulating the genes involved in lipid accumulation. Moreover, the treatments were able to protect the cytotoxic effect of steatosis, promoting the expression of survival and anti-inflammatory genes. The present study showed that the association of curcumin and andrographolide could be used as a therapeutic approach to counter high lipid content and ROS levels in steatosis liver, avoiding the possible hepatotoxic effect of curcumin. Furthermore, this study improved our understanding of the antisteatosis and hepatoprotective properties of a curcumin and andrographolide combination.

## 1. Introduction

Non-alcoholic fatty liver disease (NAFLD) is one of the most rapidly emerging liver diseases of the twenty-first century, affecting nearly two billion people worldwide [1]. More people are expected to be affected in the coming years [2], and NAFLD represents a problem of both global health and the economy [3].

NAFLD is a term that describes the histological spectrum that ranges from triglycerides (TGs) storage in the liver (steatosis), attributed as non-alcoholic fatty liver (NAFL), to nonalcoholic steatohepatitis (NASH). NASH is characterized by liver necroinflammation that results in fibrosis development [4], which can evolve into hepatocellular carcinoma (HCC), the end-stage of liver disease. Due to the increment of metabolic liver disease worldwide, by 2030, NAFLD/NASH are expected to be the most frequent reason for liver transplantation [5].

The most causative factor of NAFLD is obesity, which is strongly linked with insulin resistance (IR), as 70–80% of NAFLD patients are obese or diabetic. Other factors also contribute to the progression of NAFLD, such as genetic, epigenetic, environmental determinants, and metabolic syndrome manifestations, including hyperlipidemia, hypertension, dyslipidemia, cardiovascular disease, and type 2 diabetes mellitus [6,7].

NAFLD pathogenesis is described by the classical “two-hit” theory. The insulin resistance represents the first insult, which leads to excessive accumulation of free fatty acids (FFAs) in the liver due to the fact of reduced oxidation of FFAs resulting in the development of hepatic steatosis. The second hit is fat deposition, which leads to chronic inflammation characterized by endoplasmic reticulum stress, mitochondrial dysfunction, lipid peroxidation, oxidative stress, and cell death, leading to NASH progression and fibrosis development. Recently, the “multiple hits” model has replaced the outdated two-hit hypothesis, which suggests that multiple parallel factors act synergistically in the development and progression of NAFLD. Among myriad insults, oxidative stress is the main driver of NAFLD progression [8,9].

Oxidative stress results from the imbalance between the reactive oxygen/nitrogen species (ROS, RNS) and the antioxidant defenses. These reactive compounds are atoms or molecules that contain an unpaired electron in their outer orbital; therefore, they alter the structure and function of proteins, lipoproteins, carbohydrates, cell membranes, RNA, and DNA [10]. ROS can directly or indirectly assist the upregulation of pro-inflammatory cytokines and the activation of hepatic stellate cells leading to hepatic fibrosis development [11]. In physiological conditions, FFAs derived by the diet, de novo lipogenesis, and adipose tissue lipolysis in the liver cells are oxidized by β-oxidation or esterified into triacylglycerol and packaged into lipoproteins which are stored as lipid droplets or secreted. The accumulation of TAG in the hepatocytes alters the redox-dependent mechanisms which play part in the development of steatosis [12]. The superabundant availability of these lipotoxic lipids amplifies the production of ROS by activating mitochondrial dysfunction, endoplasmic reticulum stress, and NADPH oxidase, which further endow the disease [13].

Findings from various studies suggested that mitochondrial dysfunctions have a crucial role in the development of hepatic steatosis. Mitochondria is the main organelle that oxidizes fatty acids and generates energy. The FFAs are converted into acyl-CoA with the help of acyl-CoA synthases and enter the mitochondria with the aid of carnitine palmitoyl transferase 1 and 2. Inside the mitochondria, the β-oxidation uses four enzymes to convert acyl-CoA into acetyl CoA: acyl-CoA dehydrogenases, enoyl-CoA hydratase, L3-hydroxyacyl-CoA dehydrogenase, and 3-ketoacyl-CoAthiolase. These tricarboxylic acids cycle generates NADH and FADH_2_ which are transported to the electron transport chain and further oxidized into NAD and FAD with the generation of H_2_O. The uncontrolled transferability of FFAs to mitochondria results in deficient oxidization, which further escalates the generation of ROS [14].

To date, various drugs have been developed for the therapeutic approach to NALFD: natural farnesoid X receptor (FXR) agonist, dual peroxisome proliferator-activated receptor α-δ (PPARα-δ) agonist, and glucagon-like peptide-1 antagonists.

However, researchers are focusing more on herbal medicines due to the fact their easy accessibility and the presence of natural bioactive compounds, such as phenolic acids and flavonoids associated with their antioxidant activity [15]. Scientists believe that medicinal plants could be used for the prevention of NALFD [16]. Out of thousands of plant-based therapeutics, curcuma and andrographolide are reported to be effective in hepatoprotective roles in various in vitro and in vivo studies [17,18,19].

Curcuma, also termed turmeric, is a member of the *Zingiberaceae* family and is well known for its biological activities. Curcumin (CUR), a phytoconstituent of curcuma, is famous for anti-inflammatory, antifibrotic, immunoregulatory, hypolipidemic, antioxidant, and antitumor activities [20]. Curcumin could regulate antioxidant and pro-oxidant reactions in liver cells controlling steatosis, affecting inflammatory and hyperlipidemic reactions, and suppressing tumorigenesis, cholestasis, and fibrosis by downregulating the NF-κB pathway, CYP7A1 activity, and TGF-β signaling [21]. Preclinical studies of curcumin indicate that it could be used for the treatment of hepatobiliary diseases [22]. Whereas, the role of curcumin as an adjuvant in NAFLD patients has been evidenced by a meta-analysis approach [23], while clinical trials suggested that the use of curcumin in NAFLD, even though not proven, is promising [24].

*Andrographis paniculate* also known as the king of bitters belongs to the *Acanthaceae* family. It is an ancient ayurvedic herbal medicine used to treat several diseases. Its bioactive compound, andrographolide (ANG), mainly contributes to antidiabetic, antihyperlipidemic, anti-inflammatory, antibacterial, antiviral, antiangiogenic, antitumor, and hepatoprotective properties [19]. It also regulates redox reactions, modulates immune regulatory reactions and nitric oxide reactions, produces prostaglandin, and downregulates the NF-κB transcription factor [25,26]. Its hepatoprotective role includes repairing hepatic injury by inhibiting NF-κB signaling; affecting the signal transduction pathway MAPK, PI3K/Akt, ERK, JAK/STAT pathways; downregulating cytokines; restoring cellular permeability; inhibiting lipid peroxidation; and acting as an anti- and pro-oxidant [27].

In NAFLD, high lipid flux in hepatocytes disturbed the whole physiological balance of the liver that requires proper treatments. Nowadays, herbal medicines are frequently used to treat various kind of diseases, including NAFLD. Hence, this study was designed to evaluate the first stage of NAFLD, which is NAFL in HepG2 cell lines, and its treatment with curcumin and andrographolide separately and in association. To the best of our knowledge, this is the first study that evaluates the combined effect of curcumin and andrographolide in cellular steatosis model.

## 2. Results

### 2.1. Andrographolide–Curcumin Association Prevents Cytotoxicity in Steatosis Hepatocyte Model

**Figure 1:**
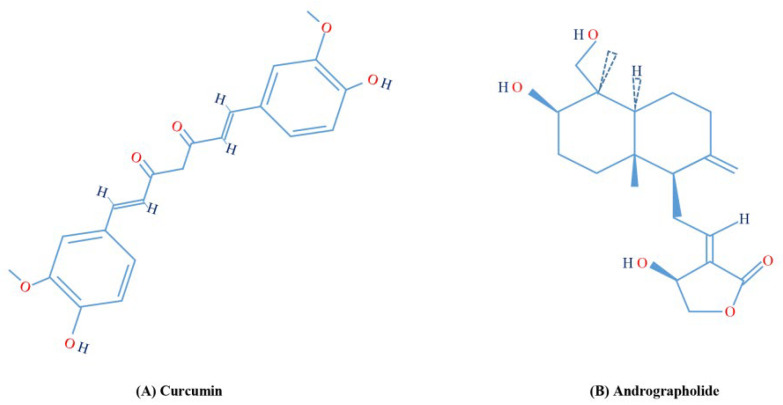
Chemical structures of (**A**) curcumin and (**B**) andrographolide.

To evaluate the effects of curcumin (Figure 1A), andrographolide (Figure 1B), and their association we used the hepatic severe steatosis model of hepatocyte culture in lipid overload-conditioned medium as described in Section 4.

**Figure 2:**
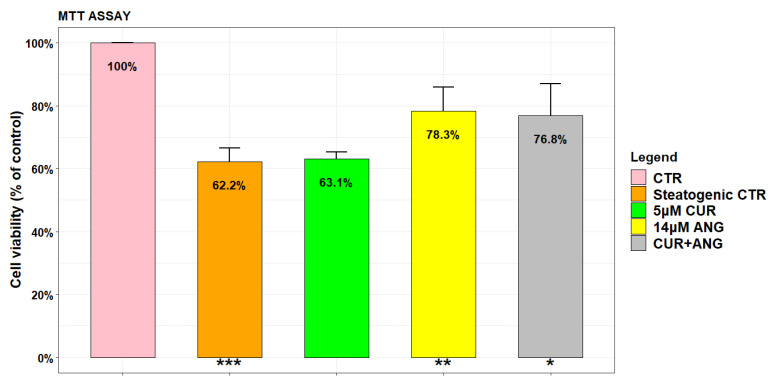
Effects of the steatogenic medium, curcumin, andrographolide, and curcumin + andrographolide on the viability of HepG2 cells. After 24 h of exposure to steatogenic medium, the cells were treated for an additional 24 h with 5 μM curcumin and 14 μM andrographolide employed alone or in association. Cell viability was assessed by the MTT assay. The results are expressed as the mean ± SD of six independent experiments. Level of significance: *** *p* < 0.001; ** 0.001 < *p* < 0.01; * 0.01 < *p* < 0.05; steatogenic control with respect to the control or treated cells with respect to the steatogenic control.

According to the literature, cells viability in steatogenic medium was lower in respect to the control in normal medium (Figure 2) [28]. The aim of our study was to evaluate the protective effects of the phytochemicals against steatosis and ROS production. For this purpose, the dosages chosen for curcumin (5 µM) and andrographolide (14 µM) were subtoxic on HepG2. It has been reported that curcumin had no significant effect on HepG2 viability in the range 5–20 µM [21] and that the IC50 for andrographolide was 40.2 µM [26,29]. The results for toxicity were confirmed by preliminary experiments on our cell line (data not shown). We revealed that the cytotoxicity of the steatosis treatment was reverted by andrographolide, suggesting its potential protective effect alone (76.3% vs. 62.2%, *p* < 0.01) and in association (75.8% vs. 62.2%, *p* < 0.05) (Figure 2).

### 2.2. Andrographolide–Curcumin Association reverted Lipid and Trygliceride Accumulation in Steatosis Hepatocyte Model

**Figure 3:**
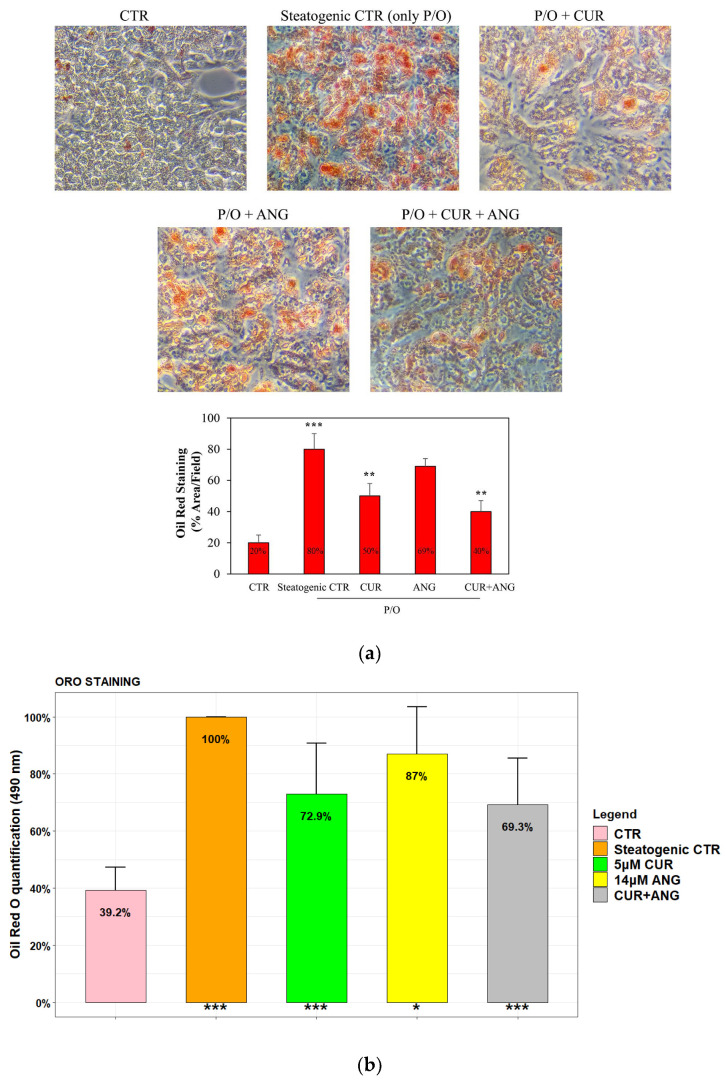

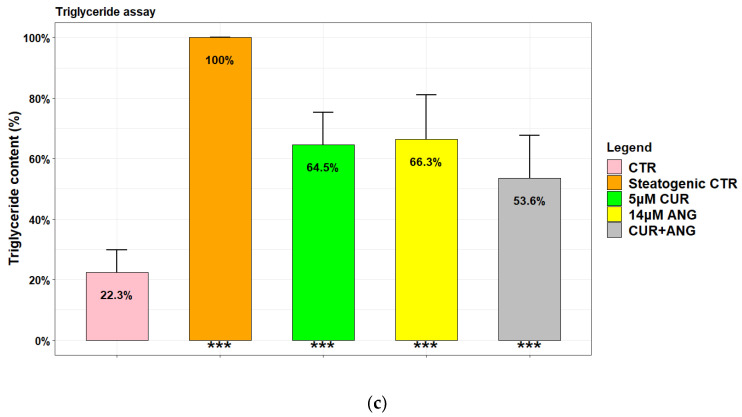
Curcumin (CUR, 5 μM), andrographolide (ANG, 14 μM), and curcumin + andrographolide reduce lipid content on HepG2 cells after steatosis induction (P/O) for 24 h as reported in Section 4. (**a**) Representative photographs showing the lipid droplets (LDs) reduction by Oil Red O (ORO) staining after the treatments (400× original magnification) and LDs quantification by ImageJ; (**b**) quantitative ORO staining measured by spectrophotometer at 490 nm reading; (**c**) intracellular TGs content quantified by spectrophotometer at 570 nm. The results are expressed as the mean ± SD of six independent experiments. Level of significance: *** *p* < 0.001; ** 0.001 < *p* < 0.01; * 0.01 < *p* < 0.05; steatogenic control with respect to the control or treated cells with respect to the steatogenic control.

To study non-alcoholic steatosis in vitro, HepG2 is the most widely used cell system model. In fact, these cells, when cultured in the presence of a specific steatogenic medium, as indicated in Section 4, accumulate lipid droplets (LDs) and modify their lipid metabolism. Oil Red O staining was used to evaluate the intracellular lipid accumulation as LDs. Microscopic examinations showed that HepG2 after treatment with P/O increased Oil Red O staining compared with the control (Figure 3a). Notably, the treatment with curcumin and andrographolide reduced the number and the size of LDs in the steatosis model alone and in association (Figure 3a). The same results were obtained by ORO quantification, which showed the reduction of absorbance (490 nm) from 100% (steatogenic control) to 72.9% (curcumin, *p* < 0.001), 87% (andrographolide, *p* < 0.05), and 69.3% (association, *p* < 0.001) (Figure 3b). The reduction in lipid accumulation was also confirmed by measuring the triglycerides (TGs) content. The treatments decreased the intracellular TGs levels to 64.5% (curcumin, *p* < 0.001) and 66.3% (andrographolide, *p* < 0.001), while the association reduced them to 53.6%, *p* < 0.001 (Figure 3c). Taken together, these data confirm that, after steatosis induction, the association reduced the LDs and TG accumulation by approximately 50%.

### 2.3. Andrographolide–Curcumin Association Reduced Intracellular ROS Production

**Figure 4:**
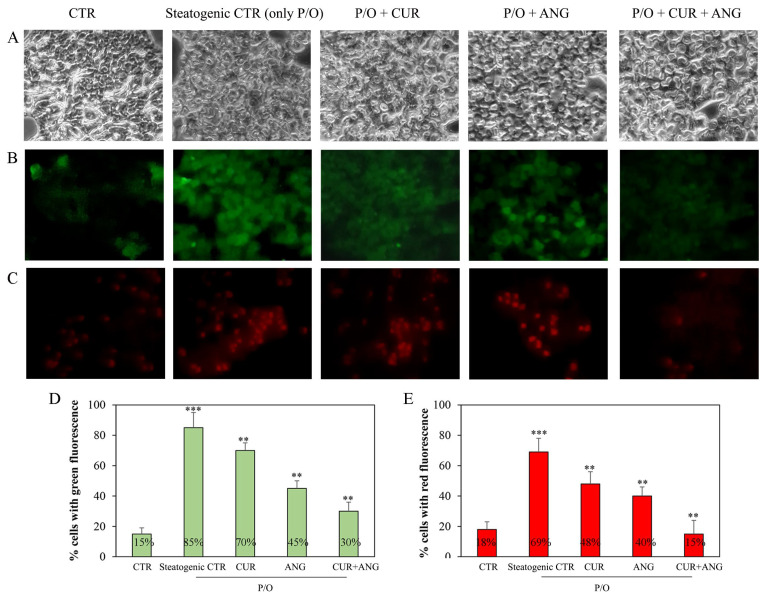
Andrographolide–curcumin association reduced intracellular ROS production on HepG2 cells after steatosis induction (P/O) for 24 h as reported in Section 4. (**A**) Cells observed under light microscopy. Intracellular ROS were detected using (**B**) H_2_DCFDA or (**C**) DHE staining as reported in Section 4. Quantification of cells with (**D**) green (H_2_DCFDA) or (**E**) red (DHE) fluorescence. Photographs were observed at 400× magnification. The results are expressed as the mean ± SD of six independent experiments. Level of significance: *** *p* < 0.001; ** 0.001 < *p* < 0.01; steatogenic control with respect to the control or treated cells with respect to the steatogenic control.

To evaluate the impact of the chosen treatments on the ROS levels in our steatosis model, we performed a fluorescence microscopy analysis by employing the redox-sensitive fluorochrome H_2_DCFDA (green fluorescence) and DHE (red fluorescence). As shown in Figure 4, the steatogenic treatment increased ROS production and both the treatment with curcumin and andrographolide seemed able to reduce partially this effect. It should be noted that the combined treatment significantly decreased the production of ROS compared to the steatogenic control (green positive: from 85% to 30%, *p* < 0.01; red positive: from 69% to 15%, *p* < 0.01).

### 2.4. Effect of Curcumin–Andrographolide Association on Insulin and Adipokine Signaling, Metabolic Pathways, Inflammatory Response, and Apoptosis

**Figure 5:**
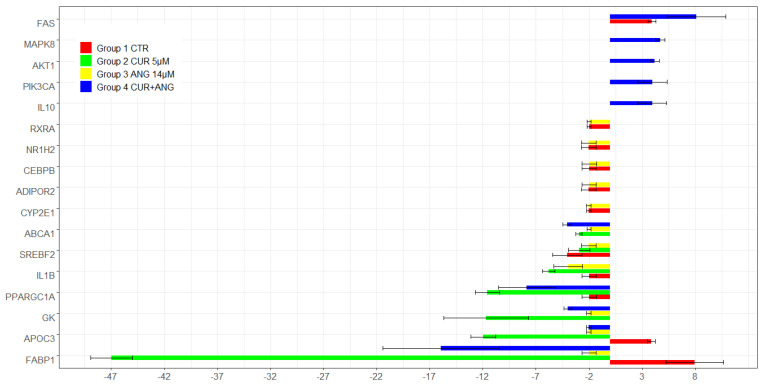
Relative changes in the fold expression of the genes involved in human fatty liver pathways (genes selected with *p*-value cutoff of 0.05). The analysis was performed in genes differentially expressed with respect to the steatosis control in dependence of the treatment with curcumin (group 2, green), andrographolide (group 3, yellow), and curcumin + andrographolide (group 4, blue). Group 1 (red) represents the HepG2 control grown in EMEM.

The microarray analysis of the genes involved in human fatty liver were performed in triplicates using HepG2 cells grown in EMEM (control, red) and HepG2 treated as reported in Section 4: curcumin (group 2, green), andrographolide (group 3, yellow), and their association (group 4, blue). The analysis was referred to the gene expression of HepG2 exposed to the steatosis medium. The comparison of the gene expression between the groups confirmed significant differences (Figure 5).

The treatment with 5 μM curcumin was able to downregulate genes involved in lipid/cholesterol metabolism and transport. The selected differentially expressed genes were identified by means of a *t*-test statistic with a *p*-value < 0.05. In detail, we found the down expression of FABP1 (40 times) and APOC3 (11.98 times), PPARGC1A and GK (11 times), ABCA1 and SRBF2 (3 times) compared to the steatosis control. Regarding genes involved in inflammatory response, IL1B expression was approximately 6 times lower in the curcumin-treated cells.

Andrographolide treatment reduced approximately two times the expression of the same genes downregulated by curcumin (FABP1, APOC3, PPARGC1A, GK, and SRBF2), while the expression of IL-1β was approximately four times lower. In addition, this treatment seemed to play the role of metabolic protection being capable to restore the normal level of genes expression upregulated after steatosis treatment, between them RXRA, NR1H2, CEBPB, ADIPOR2, and CYP2E1.

The treatment with curcumin-andrographolide in association maintained the ability to regulate in a negative manner FABP1 (15.96 times), PPARGC1A (7.85 times), GK and ABCA1 (4 times), and APOC3 (2.04 times) in respect to the steatosis control. Moreover, after the treatment with the association, the increment in the expression of the genes related to cell survival, AKT1, MAPK8, PIK3CA (four times), and FAS (eight times), and to anti-inflammatory signaling, IL10 (four times), was observed in respect to the steatosis control.

## 3. Discussion

NAFL is generally benign, whereas NASH can progress to cirrhosis, liver failure, and liver cancer. Globally, the estimated prevalence of NAFLD is 25.2%, whereas high-risk group patients, such as diabetic and obese, are 50% more prone to developing NAFLD [30]. Currently, there is no FDA-approved therapeutics available for NAFLD, although several classes of drugs for NAFLD/NASH treatment are under development, (i.e., FXR agonists and PPAR agonists) [31]. With the intrinsic association between NAFLD and metabolic alterations, lifestyle changes based on caloric restriction, a Mediterranean diet, and physical activities should be promoted for NAFLD prevention.

In recent times, research on plant-based therapeutics evidenced a curcumin and andrographolide effect in improving hepatic functions [16]. Against this background, the aim of the present paper was to investigate the activity of these phytochemicals in the experimental cellular model of NAFLD.

Curcumin (Figure 1A) is a polyphenolic natural product which exerts numerous biological activities, such as hypolipidemic, anti-inflammatory, immune-regulatory, antioxidant, and antitumor, attributed mainly to its chemical structure. Structure–activity relationship studies evidenced three important functionalities: an aromatic o-methoxy phenolic group, an alpha- and beta-unsaturated beta-diketo moiety, and a seven-carbon linker. The antioxidant activity of curcumin is due to the o-methoxyphenol group and methylenic hydrogen, acting as electron/hydrogen atom donors to reactive oxygen species [32]. We found that the treatment with curcumin reduced ROS levels in the steatosis model (Figure 4), confirming curcumin acts as a scavenger of oxygen species in this context.

On the other hand, curcumin interacts with several biomolecules through noncovalent binding and covalent binding. The aromatic and tautomeric structures which have hydrogen bonding and hydrophobicity properties, as well as their linker group, are responsible for noncovalent interactions [32]. In this way, curcumin modulates various signaling molecules with key roles in cellular signal transduction pathways pertinent to growth, differentiation, and malignant transformation and exerts potent anti-inflammatory and anticarcinogenic actions. In our experiments, the downregulation of genes involved in lipid/cholesterol metabolism and transport (such as FABP1, APOC3, PPARGC1A, GK, ABCA1, and SRBF2) induced by curcumin treatment could explain the observed reduction of steatosis and lipid content. However, it is reported that the downregulation of IL1B could be related to the anti-inflammatory response of curcumin [33,34]. Since the pleiotropic beneficial effects of curcumin are mainly related to its ability to act as a mediator of cellular redox balance [20,34], it has been listed as a natural product useful for the treatment of drug-induced liver injury or DILI [35]. However, recently, several cases of acute liver injury after consumption of curcumin as a dietary supplement have been reported [36,37], and it is still not clear when the hepatoprotective role switches to hepatotoxicity. This evidence limits the possible curcumin administration in the context of metabolic liver disease to ameliorate the lipid accumulation and the oxidative stress insult. In this perspective, the Italian Health Ministry discouraged, since July 2019, curcumin administration in cases of hepatic function alterations.

For this purpose, we evaluated the effects on lipid accumulation and ROS production by cotreatment with andrographolide, focusing our attention on its ability to prevent curcumin toxicity. It has been reported the hepatoprotective effect of andrographolide based on its antioxidant properties, which suggests its applicability in hepatic disorder treatment [38]. A rodent model of NAFLD has shown that an analog of andrographolide, isoandrographolide, ameliorates the hepatic parameters with a reduction in the transaminases and the lipid profile reducing the storage and serum lipid content [26].

Andrographolide (Figure 1B) is a labdane diterpenoid with a broad range of therapeutic applications, including anti-inflammatory and antiplatelet aggregation activities. This agent exerts anti-inflammatory effects by inhibiting nitric oxide production by macrophages, NF-kB activation, and suppressing inflammatory cytokine expression, including TNF-α, IL-1β, and IL-6. Furthermore, andrographolide acts on multiple cellular targets in the inflammatory signal transduction pathways resulting in suppressed inflammation cytokine expression, including TNF-α, IL-1β, and IL-6, and inhibits NF-kB activation. Moreover, it has been reported that andrographolide inhibits nuclear factor 4 alpha (HNF4α), one of the key regulators in liver cells that is responsible for the metabolism of xenobiotics. HNF4α possesses a ligand-binding domain for different fatty acids, favoring the interaction with coactivator PPARGC1α, involved in liver-specific genes related to the metabolism of cholesterol, bile acids, lipids, and glucose [39]. Our results show the downregulation of PPARGC1α, which could be related to the observed reduction of LDs and TG content after treatment.

Aiming to investigate the hepatoprotective effects of curcumin and andrographolide, our experiments were conducted using nontoxic doses of the phytochemicals. Notably, the treatment with andrographolide was able to revert the toxicity induced by the steatogenic medium, increasing the cell viability in respect to the steatogenic control and this effect was maintained after the treatment with the association. In this perspective, the co-administration of the two phytochemicals is attractive because curcumin mainly prevents lipid accumulation, whereas andrographolide has a cytoprotective effect, according to the described role in xenobiotic metabolism. In NAFLD, oxidative stress and pro-inflammatory cytokines play a pivot role in the pathogenesis and progression of disease. The cascade of events induced by lipid accumulation causes mitochondrial dysfunction and development of oxidative stress, which lead the disease from simple steatosis to more severe NAFLD stages. Both curcumin and andrographolide were reported to be active in the reduction of cellular ROS content [40,41]. We observed that ROS levels were abated by curcumin and, in part, by andrographolide. Notably, the effect on ROS reduction was more evident after the association treatment (Figure 4).

To investigate the molecular pathways involved in human fatty liver we used a microarray approach aimed at evaluating the expression of genes related to insulin and adipokine signaling, metabolic pathways, inflammatory response, and apoptosis. The most significative differences, shown by comparison between the treated and nontreated groups, reported in Figure 5, evidenced that the chosen treatments were effective in regulate key genes in hepatic lipid content and homeostasis. In detail, the most downregulated genes were fatty acid binding protein 1 (FABP1), glycerol kinase (GK), and peroxisome proliferator-activated receptor gamma, and coactivator 1 alpha (PPARGC1A). The marked effect of the downregulation of FABP1 after treatment with curcumin (40 times), with andrographolide (2 times), with the association (15.96 times), and GK (11, 2, and 4 times, respectively) represents, probably, the cellular mechanism of the lipid accumulation reduction observed mainly after the curcumin and association treatments. FABP1 plays a key role in the uptake and transport of fatty acid and in steatosis development, while GK is directly involved in TG synthesis [42].

The hepatoprotective effect shown by curcumin–andrographolide co-treatment, could be imputable to the observed upregulation of genes related to cell survival, such as V-AKT murine thymoma viral oncogene homolog 1 (AKT1), mitogen-activated protein kinase 8 (MAPK8), phosphoinositide-3-kinase, catalytic, alpha polypeptide (PIK3CA) (four times in respect to the steatosis control), and Fas, TNF receptor superfamily, member 6 (eight times).

Finally, the association shown an interesting activity increasing the anti-inflammatory interleukin 10 (IL 10) cytokine expression (four times in respect to the steatosis control).

## 4. Materials and Methods

### 4.1. Chemicals

*Andrographis paniculata* (95% andrographolide) and *Curcuma Longa*, (96.8% curcumin) were provided by Nuova Farmaceutica S.r.l. (Catania, Italy). Fetal bovine serum (FBS), penicillin, streptomycin, L-glutamine, 3-(4,5-dimethylthiazol-2-yl)-2,5-diphenyltetrazolium bromide (MTT), dimethyl sulfoxide (DMSO), and trypsin-EDTA were purchased from Euroclone S.p.A. (Pero, Italy). Isopropanol, ethanol, phosphate buffer saline (PBS), formaldehyde, and bovine serum albumin (BSA) were obtained from Sigma-Aldrich (Milan, Italy). Palmitic acid and oleic acid were bought from Starlab S.r.l. (Milano, Italy).

### 4.2. Cell Culture

The human hepatocellular carcinoma cell line HepG2 (HB-8065) was purchased from American Type Culture Collection (ATCC) (Manassas, VA, USA). The cells were cultured in Eagle’s Minimum Essential Medium (EMEM) (ATCC) supplemented with 10% (*v*/*v*) heat-inactivated FBS, 100 U/mL penicillin, 50 µg/mL streptomycin, and 2 mM L-glutamine. The cell cultures were maintained in flask of 75 cm^2^ at 37 °C in an incubator with a controlled humidified atmosphere at 5% CO_2_. When the HepG2 cells reached 80% confluence, they were detached from the culture flask using trypsin-EDTA (0.5 mg/mL trypsin and 0.2 mg/mL EDTA) and seeded according to the experimental conditions.

### 4.3. Steatosis Induction and Cell Treatment

In vitro steatosis model was conducted in accordance with the protocol of Campos-Espinosa and coworkers, with subtle modifications [28].

Palmitic acid (PA) and oleic acid (OA) dissolved in ethanol at 50 mM were used at the final concentrations 250 and 500 µM, respectively (1:2 ratio). To prepare the steatogenic medium, the FFAs mixture was added to EMEM supplemented with 5% BSA (vehicle), filter sterilized, and used for the steatosis induction.

All experiments were performed six times.

HepG2 cells 1 × 10^5^ were seeded in 24-well culture plate and incubated for 24 h. On the next day, the medium was replaced with the lipid overload-conditioned medium, prepared as described above. After 24 h, the medium was replaced with fresh steatogenic medium, and the cells were treated with curcumin (5 µM), andrographolide (14 µM), separately and in association, and kept overnight. Curcumin and andrographolide were dissolved in 100% DMSO as stock solutions and further diluted with bidistilled water (dH_2_O) to a desired concentration. The final concentration of DMSO in medium never exceeded 0.01% (*v*/*v*); at this concentration, DMSO had no effect on cell growth.

Cells supplemented with media containing only BSA and DMSO served as the normal control (CTR), instead cells supplemented with media containing PA, OA, BSA, and DMSO were considered as the steatogenic control. All results of the treatments with curcumin, andrographolide, and association referred to the steatogenic.

### 4.4. Cell Viability Assay

Cell viability was measured by MTT (3-(4,5-dimethylthiazol-2-yl)-2,5-diphenyltetrazolium bromide) using 1× 10^4^ cells seeded in a 96-well microplate. The next day, the cells were exposed to curcumin (5 µM), andrographolide (14 µM), and their combination for 24 h. After the treatment, 20 µL of MTT (5mg/mL PBS) was added to each well and incubated at 37 °C for 4 h. After incubation, the media were removed and 200 µL of DMSO was added to dissolve formazan crystals by constant shaking for 15–20 min. The purple formazan was quantitated by measuring the absorbance at 540 nm with a reference wavelength of 630 nm by a microplate reader (OPSYS MR, Dynex Technologies, Chantilly, VA, USA). Cell viability was measured as the percentage of the optical density (OD) values of the treated cells compared with the untreated cells as the control.

### 4.5. Oil Red O (ORO) Staining

The effect of the treatments on the HepG2 lipid content was evaluated through ORO staining (Abcam, Cambridge, UK). The cells, seeded in a 24-well plate, were fixed with 4% formaldehyde, washed with PBS, and rinsed with 60% isopropanol until completely dry. Next, the cells were stained with ORO working solution (3.5 mg/mL isopropanol) for 30 min and then washed with dH_2_O repeatedly. The pictures were obtained using a Leica DM-IRB microscope and images were acquired at the magnification of 400×. The percentage of the positive area/field at ORO staining was quantified using ImageJ as indicated, as described by Pratelli et al. [43]. To quantify the level of Oil Red O content, 100% isopropanol was added to each well before shaking at room temperature for 5 min, and the isopropanol-extracted sample was then measured at 490 nm with a microplate reader (OPSYS MR, Dynex Technologies, Chantilly, VA, USA).

### 4.6. Triglyceride Assay

The total triglyceride levels in the HepG2 cells were evaluated using a spectrophotometric commercial kit (Ab65336, Abcam, Cambridge, UK), according to the protocol of the manufacturer.

### 4.7. Measurement of Intracellular ROS

The intracellular ROS levels were detected using the cell-permeant 2′,7′-dichlorodihydrofluorescein diacetate (H_2_DCFDA) or dihydroethidium (DHE) (Molecular Probe, Life Technologies, Eugene, OR, USA) dye.

HepG2 cells (1 × 10^5^/well) were plated in 24 wells and treated as previously described. After washing with PBS, they were incubated with 1 µM H_2_DCFDA or 10 μM DHE for 15 min, in the dark, in 5% CO_2_ at 37 °C. Then, fluorochromes were removed, the cells were washed in PBS and analyzed by a Leica DMR microscope using excitation and emission wavelengths appropriate for H_2_DCFDA green fluorescence (FITC filter with λex = 485 nm and λem = 530 nm) and for DHE red fluorescence (Rodhamine filter with λex = 596 nm and λem = 620 nm). The quantity of green (H_2_DCFDA) or red (DHE) cells were counted in six different microscopic fields in each condition and expressed as percentage of the total number of cells counted under light microscopy using ImageJ.

### 4.8. RNA Isolation and Real-Time PCR Microarray

Total RNA was purified from HepG2 by miRNeasy Micro Kit (Qiagen, Hilden, Germany) and quantified using NanoDrop™ 1000 Spectrophotometer (ThermoFisher Scientific, Waltham, MA, USA).

One microgram of RNA was retro-transcribed using the RT^2^ First Strand kit (Qiagen) according to the manufacturer’s recommendations. Quantitative Real-Time PCR was performed using the predesigned RT^2^ Profiler PCR Array Human Fatty Liver (96-well format, Cat. No. 330231 PAHS-157ZC, Qiagen). The plates contained primers for 84 target genes, reported in Supplementary Materials Table S1, and for five housekeeping genes: actin, beta (ACTB), beta-2-microglobulin (B2M), glyceraldehyde-3-phosphate dehydrogenase (GAPDH), hypoxanthine phospho-ribosyl-transferase 1 (HPRT1), and ribosomal protein, large, P0 (RPLP0). In addition, each plate contained one human genomic DNA contamination control (HGDC), three positive PCR control (PCR), and three reverse transcription control (RTC).

Data are expressed as the fold change using 2^−ΔΔCt^ method referred to “control plus steatogenic medium (P/O)” as the control group. Differences among experimental groups were analyzed by Student *t*-test. The Student’s *t*-test for independent experiments was performed for testing the differences in the fold expression of genes between the experimental groups; the Bonferroni correction for multiple hypothesis testing was applied to the *t*-test and *p*-value, false-positive error probability, was used as a primary criterion for the selection of genes (*p*-value cutoff of 0.05 for statistical significance) and then the fold-change was considered as a measure of the biological significance.

The cycle threshold (ct) values were submitted to the web-based PCR Array Data Analysis software (https://geneglobe.qiagen.com/it/analyze) (accessed on: 1 December 2022) (Qiagen, Hilden, Germany).

### 4.9. Statistical Analysis

All experiments and determinations were performed six times, except the PCR microarray which was performed in triplicate. The data are represented as the mean ± SD. The statistical significance of the differences between a single group and the relative control was evaluated by a two-tailed Student’s *t*-test and adjusted *p*-values by Hommel’s method. All statistical analyses have been performed using R Statistical Software version 4.0.4 (Core Team, 2021).

## 5. Conclusions

The present study showed that curcumin and andrographolide association, at subtoxic dosages, exerts a beneficial effect on lipid storage, triglyceride content, and ROS levels in an in vitro steatosis model. The hepatoprotective activity of curcumin is controversial, with reported cases of hepatotoxicity with curcumin dietary supplementation, which lead the Italian Health Ministry to discourage its administration in subjects with liver function alterations. The goal of the co-administration of the two phytochemicals is represented by the maintenance of the beneficial effects in terms of lipid and ROS reduction together with the cytoprotective effect andrographolide mediated.

Furthermore, the data from cell viability, lipid content, ROS production, and gene expression analyses evidenced that the effect of the association on lipids was mediated mainly by curcumin, while the cytoprotective effect was principally ascribable to andrographolide. It has been reported that the FABP1 liver level represents a diagnostic marker in NAFLD [44], and circulating FABP1 have been found to be higher in type 2 diabetes mellitus patients with NAFLD [45]. We demonstrated that curcumin, alone and associated with andrographolide, reduces FABP1 expression, which has just been identified as a target for the medical treatment of fatty liver disease. Finally, the association showed an interesting anti-inflammatory effect suggesting its ability to prevent the mechanisms involved in NAFLD progression to NASH. Further studies will be conducted using cellular models of NASH to investigate the ability of curcumin–andrographolide association to prevent fibrosis development.

## Data Availability

Not applicable.

## Supplementary Materials

The following supporting information can be downloaded at: https://www.mdpi.com/article/10.3390/molecules28031261/s1, Table S1: List of 84 target genes of RT2 Profiler PCR Array (96-well format) Human Fatty Liver (Car. No. 330231 PAHS-157ZC).

## Abbreviations

ADIPOR2	Adiponectin receptor 2AKT1
V-AKT	Murine thymoma viral oncogene homolog 1
ANG	Andrographolide
APOC3	Apolipoprotein C-III
CEBPB	CCAAT/enhancer binding protein (C/EBP), beta
CYP2E1	Cytochrome P450, family, subfamily E, polypeptide 1
CYP7A1	Cytochrome P450, family 7, subfamily A, polypeptide 1
DILI	Drug-induced liver injury
CUR	Curcumin
FABP1	Fatty acid binding protein 1
FAD	Flavin adenin dinucleotide
FFAs	Free fatty acids
FAS	Fas (TNF receptor superfamily, member 6
FXR	Farnesoid X receptor
GK	Glycerol kinase
HCC	Hepatocellular carcinoma
IL 10	Interleukin 10
IR	Insulin resistance
LDs	Lipid droplets
MAPK8	Mitogen-activated protein kinase 8
NAD	Nicotinamide adenine dinucleotide
NADPH	Nicotinamide adenine dinucleotide phosphate reduced
NAFLD	Non-alcoholic fatty liver disease
NASH	Non-alcoholic steatohepatitis
NF-κB	Nuclear factor kappa B
NR1H2	Nuclear receptor subfamily 1, group H, member 2
PIK3	Phosphoinositide-3-kinase
PIK3CA	Phosphoinositide-3-kinase, catalytic, alpha polypeptide
PPAR	Peroxisome proliferator-activated receptor
PPARGC1A	Peroxisome proliferator-activated receptor gamma, coactivator 1 alpha
RNS	Reactive nitrogen species
ROS	Reactive oxygen species
RXRA	Retinoid X receptor, alpha
TGs	Triglycerides
TGF-β	Transforming grown factor beta
