# Supplementary material for: Curcumin and Andrographolide Co-Administration Safely Prevent Steatosis Induction and ROS Production in HepG2 Cell Line
Source: Molecules. 2023 Jan 27;28(3):1261. doi: 10.3390/molecules28031261 (PMC9919300; doi:10.3390/molecules28031261)
Supplement: Supplementary file 1 [file molecules-28-01261-s001.zip › molecules-2160150-supplementary.pdf]

Supplementary Table S1. List of 84 target genes of RT2 Profiler PCR Array (96-Well Format) Human Fatty Liver (Car. No. 330231 PAHS-157ZC)

1. ABCA1	31. FOXO1	61. PIK3CA
2. ACACA	32. G6PC	62. PIK3R1
3. ACADL	33. G6PD	63. PKLR
4. ACLY	34. GCK	64. PNPLA3
5. ACOX1	35. GK	65. PPA1
6. ACSL5	36. GSK3B	66. PPARA
7. ACSM3	37. HMGCR	67. PPARG
8. ADIPOR1	38. HNF4A	68. PPARGC1A
9. ADIPOR2	39. IFNG	69. PRKAA1
10. AKT1	40. IGF1	70. PTPN1
11. APOA1	41. IGFBP1	71. RBP4
12. APOB	42. IL10	72. RXRA
13. APOC3	43. IL1B	73. SCD
14. APOE	44. IL6	74. SERPINE1
15. ATP5C1	45. INSR	75. SLC27A5
16. CASP3	46. IRS1	76. SLC2A1
17. CD36	47. LDLR	77. SLC2A2
18. CEBPB	48. LEPR	78. SLC2A4
19. CNBP	49. LPL	79. SOCS3
20. CPT1A	50. MAPK1	80. SREBF1
21. CPT2	51. MAPK8	81. SREBF2
22. CYP2E1	52. MLXIPL	82. STAT3
23. CYP7A1	53. MTOR	83. TNF
24. DGAT2	54. NDUFB6	84. XBP1
25. FABP1	55. NFKB1	
26. FABP3	56. NR1H2	
27. FABP5	57. NR1H3	
28. FAS	58. NR1H4	
29. FASN	59. PCK2	
30. FOXA2	60. PDK4	
